# Self-powered, autonomous Biological Oxygen Demand biosensor for online water quality monitoring

**DOI:** 10.1016/j.snb.2017.01.019

**Published:** 2017-06

**Authors:** Grzegorz Pasternak, John Greenman, Ioannis Ieropoulos

**Affiliations:** a Bristol BioEnergy Centre, Bristol Robotics Laboratory, University of the West of England, BS16 1QY Bristol, UK; b Wroclaw University of Technology, 50-370 Wroclaw, Poland

**Keywords:** Water quality, Online monitoring, Self-powered biosensor, BOD, Energy harvesting, Microbial fuel cell

## Abstract

Standard Biological Oxygen Demand (BOD) analysis requires 5 days to complete. To date, microbial fuel cell biosensors used as an alternative method for BOD assessment requires external apparatus, which limits their use for on-line monitoring in remote, off-grid locations. In this study, a self-powered, floating biosensor was developed for online water quality monitoring. This approach eliminated the need for external apparatus and maintenance that would otherwise be required by other techniques. The biosensor was able to detect urine in freshwater and turn ON a visual and sound cues (85 dB). The energy needed to operate the biosensor was produced by the system itself with the use of electroactive microorganisms, inside microbial fuel cells. The Chemical Oxygen Demand (COD) was used as a fast method of biosensor validation. When urine concentration exceeded the lower threshold, corresponding to a COD concentration of 57.7 ± 4.8 mgO_2_ L^−1^, the biosensor turned the alarm ON. The shortest observed actuation time, required to switch ON the alarm was 61 min, when the urine concentration was 149.7 ± 1.7 mgO_2_ L^−1^. Once the sensor was switched ON, the signal was emitted until the urine organic load decreased to 15.3 ± 1.9 mgO_2_ L^−1^. When ON, the microbial fuel cell sensor produced a maximum power of 4.3 mW. When switched OFF, the biosensor produced 25.4 μW. The frequency of the signal was proportional to the concentration of urine. The observed frequencies varied between 0.01 and 0.59 Hz. This approach allowed to correlate and quantitatively detect the presence of water contamination, based on signal frequency. The sensor was operating autonomously for 5 months. This is the first report of a self-powered, autonomous device, developed for online water quality monitoring.

## Highlights

• First report of an autonomous, self-powered MFC biosensor for online monitoring.
• BOD detection capability was demonstrated with urine as the exemplar substrate.
• Frequency of the biosensor signal was dependent on contaminant concentration.
• Biosensor has been operating continuously for 150 days.
• This approach opens a new direction for developing off-grid biosensors.

## Graphical abstract

**Figure:**
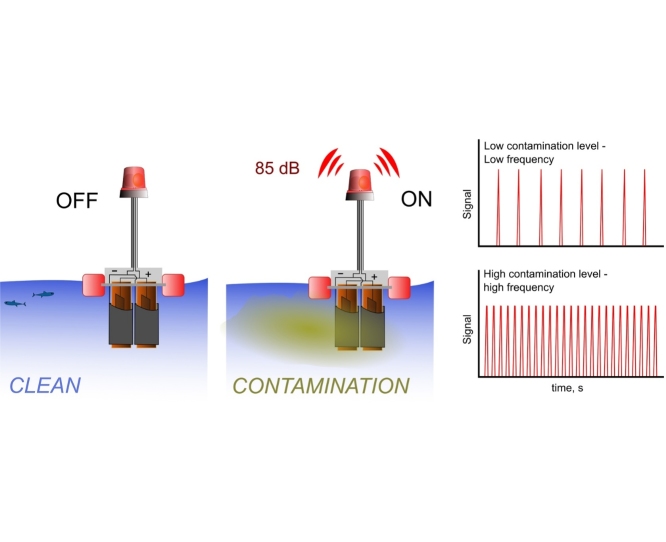

## 1 Introduction

During the past four decades, the quality of surface waters has been changing as the anthropogenic activity has led to a significant increase in organic carbon concentration. Ongoing deposition of hazardous organic substances affects the biodiversity and functioning of aqueous ecosystems [1]. Appropriate steps should be applied to minimise this negative phenomenon, including water quality monitoring. Nevertheless, conventional water quality analysis is often expensive and time consuming, as well being subject to a wide range of complex analytical methods. The majority of these methods can only be conducted offline, in well-equipped laboratories, which make such monitoring difficult in remote areas. An important part of standard water quality analysis is determining the biological oxygen demand (BOD). The principle of BOD analysis has not changed in years, and requires 5 days to be performed. Microbial fuel cell (MFC) biosensors for BOD analysis have been proposed as an alternative approach for water quality monitoring [2], although the principle has been known for almost forty years [3]. MFC biosensors, similarly as the bioreactor-based biosensors, offer the advantage of online monitoring of the biological processes and corresponding BOD [4].

A microbial fuel cell is a biological energy transducer, in which electroactive microorganisms oxidise the organic substrates and use the electrode as a terminal acceptor of electrons. In conventional single chamber MFCs, the anode is separated from the cathode by a semi-permeable membrane, which allows the transport of cations. When the external load is applied, protons and electrons combine at the cathode, which is exposed to free air, to form water. Electrical current is the by-product of biochemical oxidation of the substrate fed to the microbes in the anode [5]. Several approaches have been used to improve the performance of MFCs, including the use of novel, advanced materials [6], [7]. On the other hand, the use of ceramic separators as membranes reported in 2010 [8], has reduced the main MFC costs significantly (as low as 4.14 GBP m^−2^ [9]) since it is used as both the membrane and structural material at the same time, to, -making MFC – based technologies accessible to countries of the Developing World [10]. Moreover, the MFC technology is known for the reconfigurability of its components, that makes many practical applications possible [11].

Electricity generated in MFCs can be directly correlated with the concentration of bioavailable organic matter. Moreover, the response time of such biosensors is significantly lower, in comparison to conventional techniques. Response times in the range of minutes have been reported in the literature [12]. In a study reported by Di Lorenzo et al. [13] the response time, defined as time needed to reach 95% of the steady current, was as low as 2.8 min. The mixed bacterial populations are able to utilise a wide range of substrates, including urine [14]. Recently, MFC-based biosensors were also studied for the use in toxicity monitoring. It was shown, that the power output can be adversely correlated with the presence of organic and inorganic toxicants [15]. The sensitivity of such sensors was demonstrated for several toxic metals: when Cd^2+^ ions (0.1–100 μg L^−1^) were introduced to such sensor, the response time was 12 min [13]. In another study, Stein et al. demonstrated the sensitivity of a MFC biosensor against Ni ions equal to 0.0027 A m^−2^ mg^−1^ L^−1^ [16]. Nevertheless, MFCs are highly dynamic systems and a number of factors can influence their output signals and hence the direct “readings”. Some of these are temperature, pH and conductivity, which can affect power output and therefore signal accuracy. Several approaches have been taken to improve the correlation of MFC readings with BOD concentration and in some of these studies a determination coefficient of as high as 0.99, has been reported [17], [18]. It was also shown, that qualitative data can be extracted from the MFC output by using artificial neural networks, to identify the compounds that can be found in wastewater [19].

Although MFC biosensors offer the advantage of correlating biological activity of electroactive microorganisms with the chemical composition of the feedstock, they still require an external data logging apparatus. So far, the family of self-powered biosensors contains solutions, in which the term “self-powered” is rather referring to producing current *in situ* as a result of the reaction, but not necessarily using this generated current to power the biosensor itself [20]. For example, engineered *P. aeruginosa* cells were capable of synthesising redox mediators for current generation as a response to the presence of homoserine-lactones, and the logging of the biosensor response was performed by the external apparatus. The field of self-powered devices is currently being developed for systems consisting of flexible piezo-electric energy harvesters (nanogenerators) and these devices are mainly developed for implantable electronics in healthcare [21], [22]. An interesting approach for a self-powered device, was reported by Zloczewska et al. [23], who developed the electrochromic biosensor. The quantitative information was provided by the colour change of the prussian blue dye, as a result of current generation. Another interesting example of a self-powered biosensor, involved an enzymatic biofuel cell as the biological actuator. The biosensor proposed by Miyake et al. [24] was able to convert simple sugars like glucose or fructose into usable amounts of energy, that could be spent to power an LED diode, and the authors demonstrated its use in real biological fluids. Furthermore, it was recently shown, that a biofuel cell operating in fruit was able to produce 0.670 mW of power which was sufficient to transmit the radio signal for at least 6 h [25]. Enzymatic biosensors offer a great advantage of substrate specificity and the potential for application in monitoring sugar levels in biological tissue as well as giving relatively high power performance. They can also be integrated into high-throughput platforms, as recently demonstrated [26]. Nevertheless, the applicability of enzyme-based fuel cells for long-term online monitoring has never been demonstrated, due to the limitations of the enzyme lifetime, which varies from days to weeks [27]. Although this offers great potential in biological or food samples, the need for regenerating the enzyme(s) bound to the electrode surface makes such biosensors unsuitable for autonomous long-term operation in real environmental conditions, such as online water monitoring. In contrast, MFC biosensors are able to respond to a wide range of chemicals, offering lower substrate specificity. The bacteria present on the electrodes are able to reproduce and hence maintain the sensor’s sensitivity. Self-sustainable operation of a biosensor would offer the advantage of interpreting the sensor measurements without the need for any external power source and peripheral instrumentation.

The aim of this study was to build an autonomous, low-cost, off-grid device that would be able to detect organic contaminants, such as urine, in freshwater, and indicate their presence by emitting audio and visual signals to the surrounding environment.

## 2 Materials and methods

### 2.1 Biosensor construction

The biosensor was built using four, single chamber MFCs, electrically connected in parallel, and each MFC was made from terracotta cylinders. The ceramic cylinders served as both the cation exchange membrane and the chassis of the MFC. Each cylinder was 150 mm long, with an internal diameter of 42 mm and external diameter of 48 mm. The anode was built from carbon fibre veil with a carbon loading of 30 g m^−2^ (PRF Composite Materials, UK). Carbon veil was cut into rectangles of 1250 cm^2^ total macro surface area, folded, and wrapped around the cylinder. Plain nickel-chromium wire (Ni-Cr, 0.45 mm, Scientific Wire Company, UK) was used as the current collector and to hold the carbon veil in place. The cathode electrodes were prepared as described by Gajda et al., 2015. In brief, carbon veil was coated with a mixture of PTFE (Sigma Aldrich, UK) and activated carbon powder (G. Baldwin and Co., UK). The rectangular cathode pieces were folded and placed inside the cylinders. The hydrophobic carbon fibre veil side was exposed to air, whilst the activated carbon layer was directly in contact with the ceramic membrane. The total surface area of the cathode was 180 cm^2^ and the obtained carbon loading was approximately 60 mg cm^−2^. Such a configuration prevents the biofouling of the cathode [29].

**Fig. 1:**
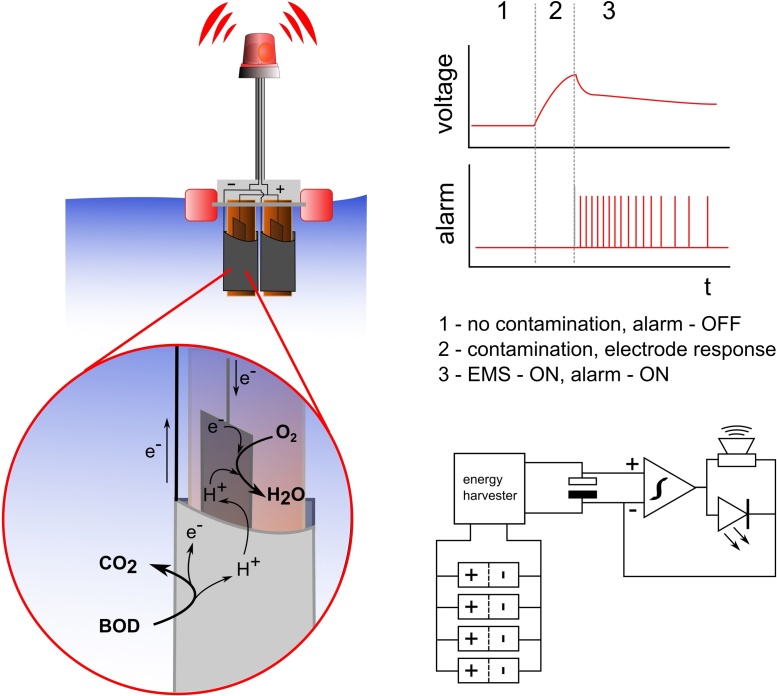
Schematic representation of the biosensor and the principle of operation. 1–biosensor operates in uncontaminated freshwater under open circuit conditions, 2–in the presence of urine, the sensor open circuit voltage increases, 3–the energy management system (EMS) switches ON, resulting in charging the capacitor up to a threshold; the audio and visual alarm is activated by the capacitor, when full, causing the latter to discharge. The system is able to repeatedly charge/discharge the capacitor.

The MFCs were attached to 3 mm thick acrylic plates and held in place by O-rings made out of rubber. Polystyrene blocks were attached to the external part of the acrylic plate, in order to allow the ceramic cylinders to be submerged in water, but leaving the top of the MFC biosensor exposed to air, when floating (Fig. 1).

The output from the four MFCs was connected to an energy management system (EMS), which consisted of: (i) energy harvester (BQ25504, Texas Instruments, USA) supplied with a variable resistor to control the maximum power point tracking (MPPT) feature; (ii) a super-capacitor and (iii) a hysteresis board. For the first 5 days of operation, a 3 F super-capacitor was used. Then, the super-capacitor was replaced by a 6800 μF capacitor until the 11th day, following which a 120 μF was connected. The hysteresis board consisted of a comparator, which allowed the super-capacitor to discharge when the voltage reached 3.1 V, and then to charge, when the voltage was down to 2.3 V. Additionally, for the period of time when the 6800 μF capacitor was connected, a comparator operating between 2.8–2.9 V was used. A red LED diode and a buzzer were connected in parallel, representing the external load. The power consumption of this load was measured prior to starting the experiment, using a separate power supply (GW-Instek PSM-3004, Taiwan).

### 2.2 Biosensor inoculation and operation

To inoculate the anodes, the MFC was placed into a container with neat human urine, supplied with pre-cultured electroactive bacteria, adapted to use urine as a fuel, from previous long-term MFC experiments, originally inoculated with activated sludge. Human urine was collected from healthy individuals with no known medical conditions. Urine was voluntarily donated and was therefore pooled prior to being collected and used in the experiments. The inoculation period lasted 5 days, during which an external load of 250 Ω was connected. This initial period allowed the biofilm to be formed on the surface of the anode. Following this, the MFC biosensor was placed into a 5L container of freshwater and connected to the EMS described above. To determine the biosensor sensitivity, threshold and response time, the water was repeatedly injected with different amounts of urine. After an initial 3-week period of operation, the MFC biosensor was placed into a new sample of the same fresh water, in order to ensure 12 complete days of anodic biofilm starvation.

### 2.3 Biosensor calibration

Once the 120 μF capacitor was connected to the EMS, the calibration of the biosensor was performed. In order to determine the frequency of the biosensor signal emitted at different power levels, the resistor connected to the energy harvester was varied. As a result of adjusting the energy harvester, different power performance levels of the biosensor were recorded. Time intervals between at least 10 individual signals emitted by the biosensor were used to calculate the frequency, (f = 1/t, where: f is frequency (Hz), t is time, s). Once the resistance of the energy harvesting circuit was set up, the sensor frequency was recorded at different concentrations of urine.

### 2.4 Water samples

Water samples used in this study were collected from the Cotswold Water Park (UK) and analysed for chemical oxygen demand (COD), pH and conductivity, during the experimental period. Urine concentration was calculated by subtracting the COD of the freshwater from the total COD. The standard COD test kit was used along with an MD200 colorimeter (Camlab, UK), according to the manufacturer’s instructions (Camlab, UK).

### 2.5 Polarisation experiments

To characterise the power performance of the MFC biosensor, polarisation experiments were performed. The experiments were performed using the resistostat – an automated variable resistor system [30]. The polarisation was done by using the set of resistors ranging from 1 MΩ to 3.75 Ω. Each value was connected to the electrical output of the MFC biosensor for a period of 5 min. The polarisation was done to determine the MFC characteristics under two different operating conditions: (i) – high concentration of nutrients, (ii) – low concentration of nutrients and no signal emitted by the sensor (below detection limit).

### 2.6 Data logging and processing

The performance of the biosensor was recorded using an Agilent 34972A Data Acquisition unit (Agilent Technologies, USA). The data logging sample rate was set to 3 min. The MFC biosensor signal was recorded using a Picolog ADC-24 Data Logger (Pico Technologies, UK), with a sampling rate of 0.5 s. The power performance (P) in Watts (W) was calculated according to the following equation: P = I x V, where I is current (A) and V is voltage (V). Experimental data were processed using Microsoft Excel 2010 and plotted by GraphPad Prism 5 software.

## 3 Results and discussion

### 3.1 Biosensor construction

The operational principle of the MFC biosensor is shown on Fig. 1. The energy, required by the electrical devices connected to the biosensor, was produced by electrically connecting four MFCs in parallel. When urine was added to freshwater, the open circuit voltage (OCV) of the MFC biosensor had increased. Increased voltage resulted in turning ON the energy harvester and closing the circuit. The electricity generated by the MFCs within the biosensor setup was used to charge the capacitors, which were controlled by the EMS. Therefore, when the urine concentration in water had reached the appropriate sensitivity threshold, the capacitors supplied the energy to power the warning light and the sound alarm (85 dB, according to the manufacturer).

### 3.2 Performance characterisation

**Fig. 2:**
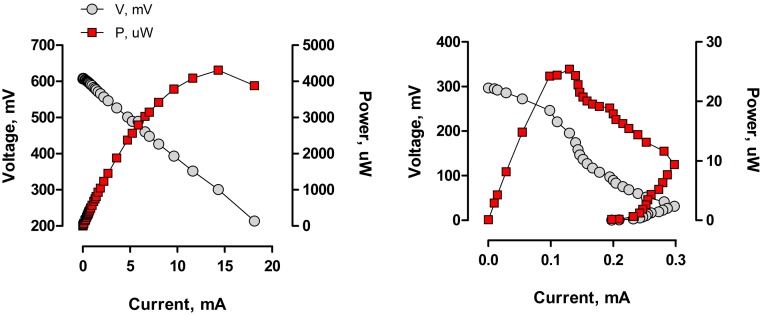
Polarisation and power curves for the MFC biosensor, fed on the highest (left) and the lowest (right) organic loads.

To validate the performance of the electrodes, polarisation experiments were performed using high (6.84 ± 0.05 gO_2_ L^−1^) and low (15.3 ± 1.9 mgO_2_ L^−1^) organic load concentration in urine as the feedstock. The first test was performed after 5 days of operation. The results shown in Fig. 2 indicate that after only 5 days since inoculation, the biofilm had established well at the electrode surface. The power performance of the MFC biosensor reached 4.31 mW, and the maximum current was 18.1 mA. The OCV reached 608 mV and the biosensor internal resistance was determined to be 21 Ω for the collective, i.e. 84 Ω for the individual MFCs. The internal resistance (R_int_) was estimated based on the maximum power point (MPP), which occurs when the R_int_ is equal to external resistance [31]. The power performance achieved by the individual MFCs was higher in comparison to the other known terracotta-based MFCs [28], [32], [33]. The polarisation curves revealed low internal losses, except of course for the low concentration, which was expected to result in a distorted power curve.

The lower organic load concentration test was performed after 61 days of operation, when the sensor was fed on freshwater. The polarisation was done when the performance of the MFC biosensor decreased and the alarm signal was no longer emitted. The OCV value was of 297 mV, the current reached 297.7 μA, whilst the maximum power was 25.4 μW. At this lower concentration of urine, an overshoot phenomenon was observed. The overshoot effect is common for the underperforming MFCs and its presence may be affected by several factors [31], [34]. In this study, most likely it was the result of insufficient feedstock supply.

The polarisation experiments revealed that the MFC biosensor was able to generate electricity at varying urine concentrations. The lower power threshold determined during the experiment – 25.4 μW was insufficient to maintain the operation of the EMS and to generate any warning signals.

### 3.3 Biosensor operation

**Fig. 3:**
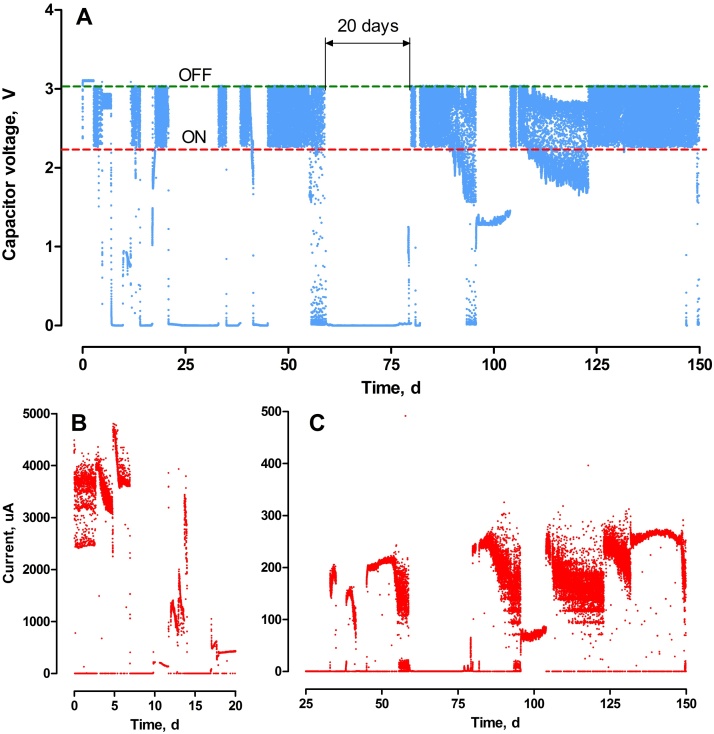
Real-time monitoring of the MFC biosensor signal and performance. A – Capacitor voltage, B – Sensor adjustment and calibration period, C – sensor operational period. Each increase in the capacitor voltage above the ‘ON’ threshold (from 0 V to 2.3 V) corresponds to the addition of urine.

In the initial 20-day period, various operating conditions were tested (Fig. 3). During the first 5 days, the sensor was supplied with a large 3 F super-capacitor. This capacitor was appropriate only when neat (undiluted) urine was used as the fuel. The observed signal frequency was 8.3 × 10^−4^ Hz (every 20 min). A smaller, 6800 μF capacitor was used between the 5th and 11th day of operation, along with a comparator working within a narrower voltage range (charging the capacitor at 2.8 V and discharging at 2.9 V). Although in this configuration the sensor was emitting a signal with a frequency of 0.5 Hz (every 2 s), a significant current leakage was observed (data not shown). Therefore, to increase the sensitivity of the biosensor at the lower urine concentration, a 120 μF capacitor along with a comparator operating at 2.3–3.1 V were used. The power consumed by the load (LED and buzzer) was 13.0 mW and 4.0 mW for 3.1 V and 2.3 V, respectively.

**Fig. 4:**
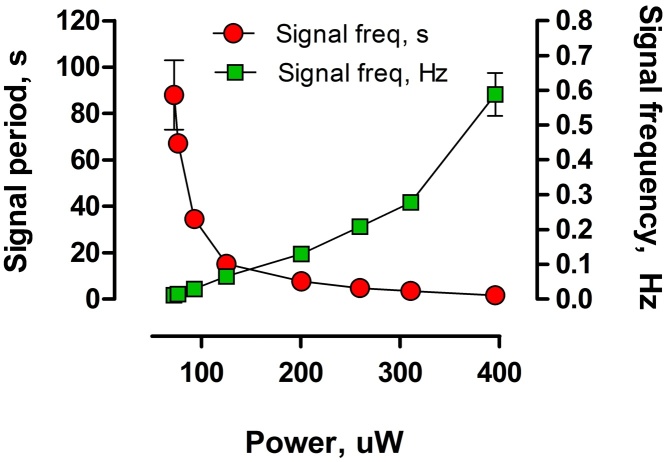
Calibration curve of the emitted signal, measured for different power levels of the sensor.

Once the configuration of the system had established, the sensor was adjusted by controlling the variable resistor, connected to the energy harvester. Varying the resistance value allowed to control the MPPT feature of the harvester. Thus, the harvester was programmed to remain under closed circuit when between 50 and 90% of the OCV. The resulting power output observed in this period varied and the power levels were reaching 603.1 μW. Adjustment of the MPPT feature was performed between the 11^th^ and 14^th^ day of operation. The data from this period was used to produce a calibration curve (Fig. 4), which showed a proportional relationship between the signal frequency and the power generated by the MFCs. The highest frequency of observed, stable signal was equal to 0.59 Hz (for 395.7 μW). When the MFCs generated 75.5 μW, the frequency was 0.01 Hz. These values corresponded to a signal emitted every 1.7 and 88 s, respectively.

The influence of external load on MFC biosensors was investigated by Stein et al. [35]. They showed that lower external resistance has got a positive effect on the sensitivity of a biosensor, but also leads to a decreased recovery time for the anode electrode. Since the biosensor reported herein was controlled by the EMS, its sensitivity and response time were only dependent on urine concentration and background/residual COD concentration. Therefore, assuming that the biofilm established in the MFC biosensor, will be subject to long starvation periods, the final value of the external load was set to 75% of the open circuit voltage. This approach also allowed to decrease the rate of bacterial catabolism. As a result, the signal transmission was sustained for longer, when for example compared with the lower external resistances. This is particularly important when the concentration of contaminant is low and the rate of its biotic and abiotic degradation in the environment is high. Similarly, when a higher signal frequency would be desired, the external resistance of the sensor could be set to a lower value.

The time, in which the signal was emitted by the biosensor varied between 2 and 10 days in the first 2 months of operation and was mainly dependent on urine concentration. During these periods, up to tens of thousands of charge/discharge cycles were recorded. The longest signal emission was observed for a urine concentration of 149.7 ± 1.7 mgO_2_ L^−1^. A stable signal, starting from 45^th^ day of operation and recorded for 10 days was followed by a series of intermittent signals, observed by the drop of the capacitor voltage below 2.3 V (Fig. 3). When urine was consumed, and its concentration decreased to the sensitivity threshold, the closed circuit voltage was not sufficient to maintain the harvester in operation. Once the sensor was brought back to open circuit mode, the OCV of the MFCs increased resulting in another actuating cycle of the signal. This suggests that electroactive bacteria were able to adapt to decreasing concentrations of urine. Adapting microorganisms to low concentration of carbon sources allows the bacteria to save the energy required for new regulatory pathways [36] and can result in higher affinity for the rate limiting substrate. Thus, it is possible to induce this physiological training in the biofilm and part of our further work on bacterial adaptation to low concentrations of urine, may lead to improved biosensor sensitivity.

**Fig. 5:**
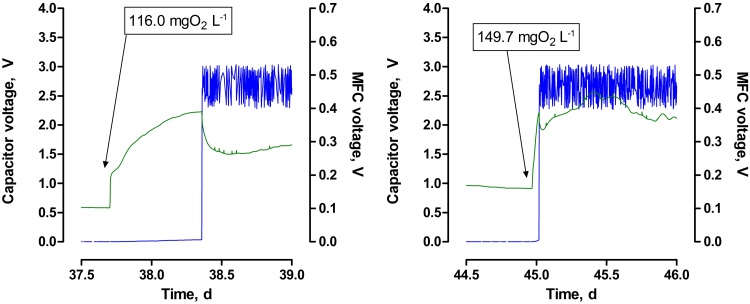
Response and actuation time for the biosensor fed on different concentrations of urine.

The recorded response time recorded for MFC potential was 3 min, when urine was added to water. However, the minimum voltage, required by the harvester to run the circuit, was 384.5 ± 6.58 mV (Fig. 5). Therefore, the actuation time of the MFC biosensor varied, and was dependent mainly on urine concentration, but also on the OCV before the urine was added. The actuation time for two different urine concentrations is shown in Fig. 5. When the water was supplemented with 116.0 ± 4.0 mgO_2_ L^−1^ of urine, the actuation time was equal to 16.80 h. When the urine concentration reached 149.7 ± 1.7 mgO_2_ L^−1^ the actuation time was only 1.15 h.

The short response time is a known feature of MFC-based biosensors. The response time for MFC-BOD biosensors varies between 3 and 80 min [12]. Although the time required to charge the capacitors and emit the signal by the biosensor described in this study, was varying from one hour to one day, it was nevertheless shown that relatively low water contamination could be detected by this autonomous system.

**Fig. 6:**
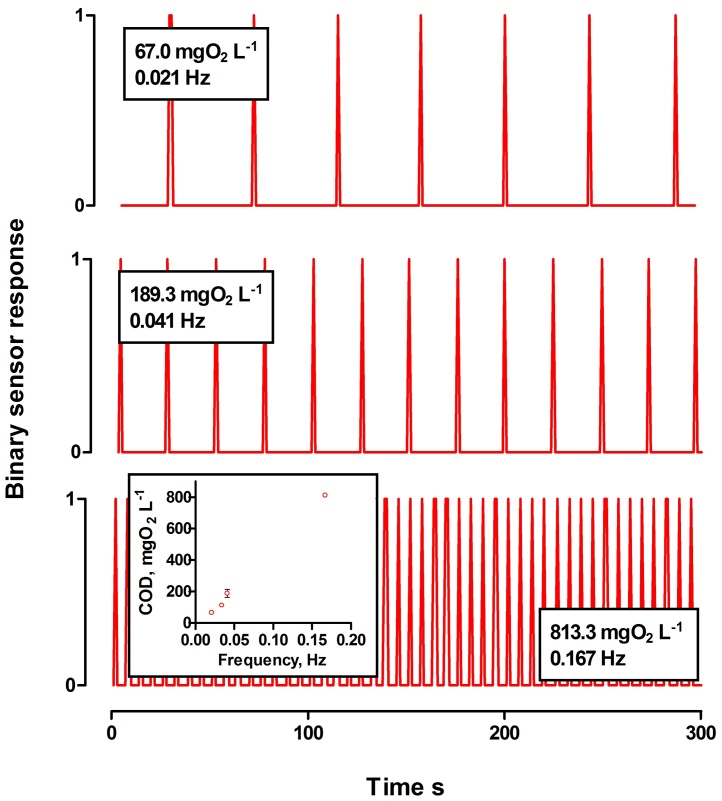
The signal emitted from the biosensor when different urine concentrations were present in freshwater. Each peak corresponds to a sound and light signal. Although the operating voltage range for the capacitor was between 2.3–3.1 V, the results are shown as a binary system response, where the frequency of the recorded signal, reflects the organic load concentration. The scatterplot representation of the frequency and organic load dependence is given in the inset (data represent mean ± SD).

Power produced by the MFCs is dependent on the concentration of the biofuel [3], [37], [38], which also applies to enzymatic fuel cells [24], [39]. To validate the dependence of the response on the concentration of urine, the signal was recorded across different set levels of urine contamination, whilst a constant external resistance was connected to the EMS (Fig. 6). At the lower threshold of sensitivity, the signal frequency was of 0.021 Hz, in the presence of urine, with an organic load of 67.0 ± 2.16 gO_2_ L^−1^. Increased concentrations of urine resulted in higher frequencies of recorded signal, as presented on Fig. 6. Therefore, the signal frequency alone can be interpreted as sensory information, to distinguish the water contamination level. Application of the sound and light signals simply expands the range of environments that this technology could be applied in. Moreover, it is known that MFCs are able to operate for more than 5 years with no servicing [17], [40]. Therefore, an autonomous online monitoring BOD biosensor such as the one presented herewith, may be helpful in assessing the water quality in areas, where no electricity is provided or low maintenance is required.

## 4 Conclusions

We developed an autonomous, self-powered biosensor for online water monitoring. Urine, which is a mixture of organic and inorganic compounds was used to demonstrate its COD/BOD biosensing capabilities. The sensor was constructed from microbial fuel cells and an energy management system. In the presence of urine, the sensor was able to switch ON the sound and light cues, which lasted for at least 2 days and in the long run, the sensor was successfully operated for 150 days. The interpretation of the signal is intuitive, either based on its frequency or simply its presence. Thus a fully automated biosensor is envisaged in off-grid areas, such as lakes or water sources, where it can be a part of an early warning system, and the first steps in this direction have been demonstrated with the reported lab scale system. This report opens up new directions for developing autonomous MFC biosensors.
